# Expression profiling of repetitive elements by melting temperature analysis: variation in HERV-W *gag *expression across human individuals and tissues

**DOI:** 10.1186/1471-2164-10-532

**Published:** 2009-11-17

**Authors:** Christoffer Nellåker, Fang Li, Fredrik Uhrzander, Joanna Tyrcha, Håkan Karlsson

**Affiliations:** 1 Department of Neuroscience, Karolinska Institutet, Retzius väg 8 B2:5, 17177 Stockholm, Sweden; 2 Mathematical Statistics, Stockholms Universitet, Kräftriket Hus 6, 106 91 Stockholm, Sweden

## Abstract

**Background:**

Human endogenous retroviruses (HERV) constitute approximately 8% of the human genome and have long been considered "junk". The sheer number and repetitive nature of these elements make studies of their expression methodologically challenging. Hence, little is known of transcription of genomic regions harboring such elements.

**Results:**

Applying a recently developed technique for obtaining high resolution melting temperature data, we examined the frequency distributions of HERV-W *gag *element into 13 Tm categories in human tissues. Transcripts containing HERV-W *gag *sequences were expressed in non-random patterns with extensive variations in the expression between both tissues, including different brain regions, and individuals. Furthermore, the patterns of such transcripts varied more between individuals in brain regions than other tissues.

**Conclusion:**

Thus, regulated expression of non-coding regions of the human genome appears to include the HERV-W family of repetitive elements. Although it remains to be established whether such expression patterns represent leakage from transcription of functional regions or specific transcription, the current approach proves itself useful for studying detailed expression patterns of repetitive regions.

## Background

The human genome contains approximately 3 billion base pairs. Only approximately 2% of these encode the proteins which carry out almost all of the known cellular functions [1]. The remaining 98% of the human genome has, by and large, been considered "junk" DNA. During recent years, data from tiling arrays and large-scale sequencing of cDNAs indicates that large amounts of the junk DNA is transcribed, not only intronic DNA as parts of unprocessed pre-mRNAs, but as tightly regulated cell-specific transcripts from both strands in intronic as well as intergenic regions (for a review see[2]). Approximately 8% of our genome consists of sequences classified as human endogenous retroviruses (HERV), ancient remnants of retroviral integrations and their subsequent expansions in the genomes of our ancestors [3]. These HERV elements have degenerated over millions of year of evolution and can, with few exceptions, no longer encode complete proteins let alone engender infectious viral particles [4-11]. Since genomic regions harboring repetitive elements, including HERV, are for methodological reasons usually excluded from array-based large-scale expression studies, their potential transcriptional activities and biological relevance remain largely uncharacterized, despite the numerous observations of their differential expression in human diseases [12-18].

As for the rest of the genome, evidence is mounting that genomic regions containing HERV elements are transcriptionally active in human tissues, see [10,19,20] and related references. Systematic studies of HERV expression patterns across tissues have been limited to quantifying expression of single elements or collective expression of entire HERV families [5,7,21-23]. These studies suggest that levels of transcripts encoding HERV elements vary between tissues [21-24]. The repetitive nature of HERVs makes designing assays that specifically detect individual elements difficult and carefully optimized probe assays are usually required for specificity [25]. The sheer number of members in the different HERV families [26] makes it a prohibitively expensive and sample consuming to determine expression patterns of more than a few elements within the different HERV families. Therefore, a systematic evaluation of expression patterns of individual members of a HERV family across tissues and individuals has not previously been performed. We have previously used analysis of melting temperatures (Tm) in semi-quantitative PCRs (qPCR) as a proxy marker of sequence differences between amplicons generated by primers targeted toward the HERV-W family [27,28]. By manually categorizing melting temperatures into 3-4 distinct groups, cell type specific expression patterns of HERV-W elements in human cell lines were observed [27]. Sequencing and mapping of PCR-products representative of these different groups indicated that a number of genomic loci were transcriptionally active in these cells. Cell-type specific changes in the expression patterns following serum deprivation or influenza A/WSN/33 virus infection indicate regulated expression of several of these loci.

We recently refined the Tm-assay by using a molecular beacon as an internal control for temperature variations over the heat-block in the thermocycler and an automated analysis program for more precise and unbiased Tm acquisition. By this approach, the resolution was improved by a factor of ten [29] allowing acquisition of more detailed data. These data can subsequently be analyzed by application of mixture models analysis for an objective determination of minimum numbers of sequences represented by the detected Tms and the frequency distributions of these Tms [30].

In the present study we applied these techniques to determine expression patterns of transcripts containing HERV-W *gag *sequences with unprecedented resolution, however, absolute expression level differences between tissues were not examined. We report expression pattern profiling of transcripts containing HERV-W *gag *sequences in human primary fibroblast cultures and tissues including; blood, thymus, spleen, ovary, testis, liver, and regions of the brain.

## Results

**Figure 1:**
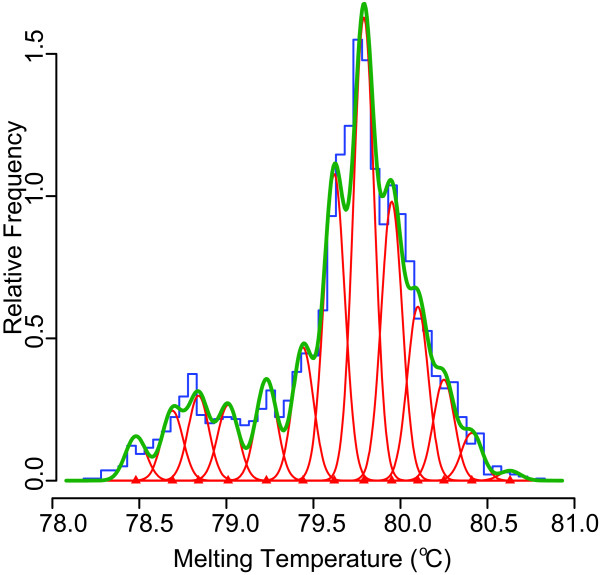
**Mixture model analysis of 2775 Tm data points for HERV-W *gag *amplicons**. Illustration of the model constructed using mixture models analysis and determined to be optimal according to Akaikes' information criterion with 13 Tm categories. Blue line represents the stratified Tm data from the 2775 Tm data points. Red Gaussian curves show each of the component Tms, their mixing proportion and the measuring error spread (with red triangle on the base line showing the precise Tm of each category). Green line shows the summed model that is fitted to the stratified data (blue line).

We applied the Gaussian curve fitting and temperature normalization with a molecular beacon method previously described [29] to human tissue samples. When we had accumulated 2775 individual Tm observations from a wide range of human tissues we constructed a model (as described in [30]) of the minimum number of Tm categories required to explain the spread of the data (based on that the SD of recording a single Tm in the instrument was 0.06°C [29]). The mixture model Tm-analysis technique predicted a model of mixture proportions into 13 Tm categories. This number of categories was found to be optimal according to Akaike's information criterion (Figure 1) as previously described [30]. With this model we proceeded to fit sets of individual Tms recorded from tissues to the model consisting of the 13 Tm categories.

**Figure 2:**
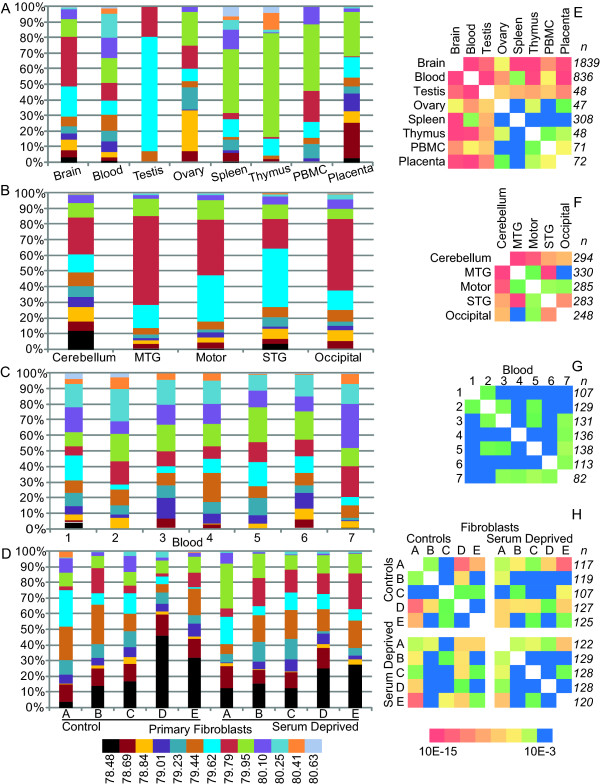
**Frequency distributions of Tm categories of transcripts containing HERV-W *gag *elements in human tissues**. Panels A, B, C and D illustrate the frequency distributions of the Tm categories in A: human tissues, B: brain region averages from three individuals, C: whole blood samples from 7 individuals and D: primary fibroblast cultures control and serum deprived. Panels E, F, G and H are heat-maps of p-values obtained from Chi-square analyses of the corresponding frequency distribution diagrams. Blue is considered not significant with p > 0.001, p-values decrease as the color moves towards red (p < 10^-15^). The number of Tm recordings per tissue is designated to the right of the heat-maps, n.

Data points were subsequently plotted in frequency distribution histograms visualizing expression patterns in different tissue and cell types, Figure 2A. To investigate if expression patterns of transcripts containing HERV-W *gag *sequences differ between regions of the human brain, four regions of cerebral cortex and the cerebellum were analyzed. Frequency histograms presenting these data are presented in Figure 2B. To investigate the extent of inter-individual variation of expression of transcripts containing HERV-W *gag*, we also analyzed whole blood obtained from seven blood-donors, Figure 2C. To try to distinguish the genetic from the environmental components of the expression patterns of HERV-W *gag *elements, we employed cultures of human primary fibroblasts from healthy individuals and subjected them to serum deprivation. This was also intended as a high resolution follow up from our previous work on the environmental influence on expression pattern of HERV-W *gag *elements[27], Figure 2D.

Heat-maps of p-values obtained from pair-wise Chi-square comparisons of expression patterns in the different tissues are illustrated in Figure 2E-H. All comparisons, except that between spleen and PBMC, suggest significantly differing patterns of transcripts containing HERV-W sequences in human tissues, Figure 2E. Similarly, expression of such transcripts also differed between the investigated regions of the brain (Figure 2F). Blood samples from the different individuals, however, exhibited a far more homogenous expression pattern (Figure 2G). We observed that primary fibroblast cultures exhibited a higher degree of variation between individuals than the less cytologically defined whole blood samples (though not from the same individuals, Figure 2H). The patterns became more similar between individuals in response to serum deprivation, in line with the hypothesis that HERV-W *gag *expression correlates to specific expression changes.

**Figure 3:**
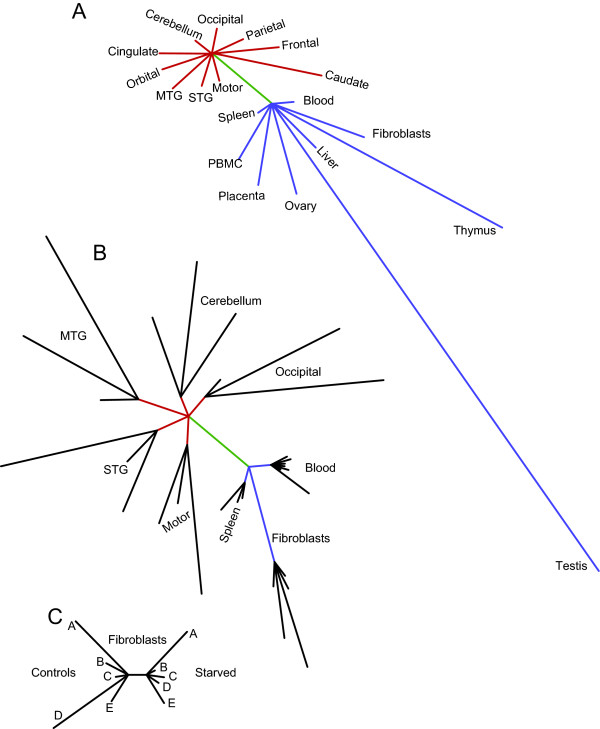
**Distances between all tissues studied**. To illustrate the degree of variation in HERV-W *gag *expression patterns between tissue types and individuals, distances (mean squared differences of mixing proportions) were calculated. The average frequency distributions in all non-CNS and CNS tissues were used as references, which were calculated by adding all corresponding tissue data together. Distances from the references to individual tissues were defined as the sums of squares of mixing proportion differences, Tm-category by Tm-category. Panel A shows the variation in HERV-W *gag *expression pattern between averages of tissues. The red lines indicate distances between each CNS tissue to the average frequency distribution of all CNS tissues. The blue lines indicate distances between each non-CNS tissue to the average of all non-CNS tissues. The green line connecting the two nodes represents the distance between average CNS and non-CNS tissue frequency distributions. Panel B shows variations in HERV-W *gag *expression pattern between individuals and tissues for the subset of the data where individual tissue samples were available. The green, red and blue lines are the same as in Panel A. The distance to each individual is calculated relative to the average frequency distribution of each tissue. Panel C shows the variation in HERV-W *gag *expression pattern between human primary fibroblast cultures and the variation after serum deprivation. The two nodes represent the average pattern of expression in the five individual cultures under control and serum deprived culture conditions.

To illustrate the degree of variation in expression patterns between samples, we calculated the relative distances (summed square differences of Tm-categories) between single tissues and averages of tissues (Figure 3). Of all tissues tested, thymus and testis exhibited the most differing expression patterns of transcripts containing HERV-W *gag *sequences (Figure 3A). Indeed, approximately 70% of all transcripts detected in cDNA from testis or thymus matched single Tm-categories, whereas the distribution was more even between Tm categories in other tissues. The distances calculated between individual blood samples were smaller than those observed between different tissues or brain regions (Figure 3B). Patterns of HERV-W *gag *expression between individuals varied more in brain regions than in spleen tissue samples, whole blood or primary fibroblast cultures. Taken together, variation in expression patterns in primary fibroblast cultures were reduced in response to serum deprivation (Figure 3C).

**Figure 4:**
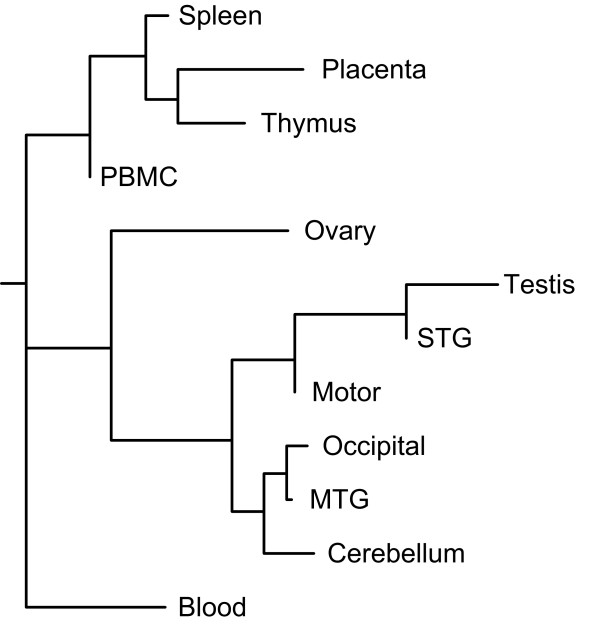
**Dendrogram from neighbor-joining of Pearson correlation coefficients between tissues (average mixing proportions)**.

We next looked at similarities in expression patterns between tissues by constructing neighbor-joining trees based on correlations in expression patterns between samples. As can be seen from Figure 4, brain regions were more closely correlated to each other than to other tissues. Similarly, placenta, spleen, thymus and PBMC were also more closely correlated to each other than to the brain regions or gonads. Blood samples had a relatively even distribution of mixing proportions into the 13 Tm categories and are consequently poorly correlated to all other tissue samples.

## Discussion

We here report expression patterns of transcripts containing HERV-W *gag *sequences in human tissues. Transcripts containing HERV sequences in general, along with other repetitive regions of the genome are generally not prioritized in human genomics studies. Due to the difficulty in finding specific primers and probe sequences, cross-reactions between different elements would render regular PCR or array based techniques ineffective for distinguishing individual members within a HERV-family. Tm-analysis has the advantage of detecting variations in the sequence of an amplified region, predicting the least number of different sequences required to account for a set of Tm data. It can, however, never predict more than the *minimum *number of different sequences as different sequences can have indistinguishable Tms [29]. Expression pattern variations between such sequences can therefore remain undetected. However, with Tm-analysis it is possible to screen samples for differences in expression patterns requiring sequencing of only a limited number of products within a Tm category of interest. These can subsequently be differentially analyzed by more specific assays, as exemplified in [28] or can be sequenced selectively based on Tm. The approach used allows a higher resolution of repetitive sequence analysis without the expense of large scale sequencing. The evaluation of cost versus gain in information of the method used here as compared to the high throughput sequencing data produced in next generation sequencing technologies is a subject for future studies.

The current findings illustrate part of the hidden complexity of the human transcriptome. We can conclude that expression of HERV-W *gag *sequences varies between human tissues systematically and consequently in a non-random fashion. Tissue type and cell composition appear to be a larger determinant of the expression profile of such transcripts than the individual from which the tissues were obtained. For instance the expression profile of HERV-W *gag *containing transcripts in testis, a pooled sample of, according to the manufacturer, 53 individuals contained only 3 categories of 13 possible represented (an unlikely finding if expression was random).

Neighbor-joining of the Pearson correlation coefficients resulted in a dendrogram which groups brain regions and gonads together and tissues rich in cells of the immune system together. This dendrogram resembles similar constructs built from coding transcript expression data [31], indicating that transcripts containing HERV-W *gag *elements vary between tissues similarly to such transcripts. Due to the limited number of data points the tree structure is not stable and is hence only an indication of similarities. Previous studies on transcription in different tissues, including different brain regions, suggest that functional specialization is reflected at the level of transcription [32-34]. Based on the clustering of tissues in Figure 4, it appears as if this extends also to the transcription of HERV-W elements.

We observed that the degree of differences in expression patterns of HERV-W *gag *elements between individuals was not constant across tissues and cells. Expression patterns of coding transcripts are known to vary in whole blood samples depending on age, gender, time of day and health status [35], despite this whole blood exhibited the most homogenous expression pattern of the tissues investigated here. Interestingly, expression patterns in brain tissue exhibited the largest variation across individuals of all tissues examined. Since spleen samples obtained from the same individuals, displayed far less variation, these findings cannot be attributed to *post-mortem *or nucleic acid purification artifacts. Indeed, Franz and coworkers [36] reported that death caused exaggerated homogeneity in expression profiles of coding transcripts in human brain. Human fibroblasts lines from different individuals were maintained for 3-5 passages under identical conditions, yet differences between individuals remained, regardless of treatment. Thus, in addition to environmental cues, genetic or epigenetic components [37] appear to contribute to the HERV-W *gag *expression patterns.

Whether this tissue specific transcription is a consequence of specific mechanisms or reflects transcriptional leakage from transcription of coding regions remains to be established. Indeed, putative promoter structures (i.e. long terminal repeat regions) in the HERV-W and other families are active in human cells [20,24]. Recent studies indicate that tens of thousands of human transcripts are initiated at retroviral promoters [38]. Furthermore, Gogvadze and coworkers [39] recently reported functions of HERV-K transcripts in regulation of gene expression.

## Conclusion

Our current findings that human tissues harbor patterned and extensive expression of genomic loci containing HERV-W elements, suggests that functionality for such transcripts cannot be ruled out. Finally, the methodological approach used here proves itself useful for detailed analyses of transcripts originating from repetitive regions. The potential applications for this method would be to examine expression pattern alterations of repetitive sequences in disease states. Detailed expression patterns of repetitive elements have yet to be explored in cancers or neurodegenerative diseases where specific or total expression changes have been documented. This methodological approach might be useful for determining whether such quantitative changes originate from specific loci or from global expression changes of repetitive elements.

## Methods

### Tissue isolation and culture

To establish fibroblast culture, one 2-mm diameter cutaneous biopsies were taken from five volunteers following informed consent. Samples were ground and placed in a 6-well plate under a sterile glass cover slip. Fibroblasts were subsequently cultured in DMEM/20% FCS/non essential amino acids/penicillin/streptomycin/sodium pyruvate (Invitrogen, Carlsbad, CA, USA) in a humidified 37 C, 5% CO incubator. The study was approved by the regional ethics committee (04-273/1; 2006/637-32). Cells were used between passages 3 and 5.

### RNA preparation and first strand synthesis

Human oligo d(T)-primed cDNA samples generated from ovary, thymus, testis and placenta were purchased from Ambion (Austin, TX, USA). Human whole blood samples were volunteered anonymously from blood donors. Samples of human spleen, cerebellum, frontal, orbital, motor, occipital and parietal cortices as well as cortex from the medial temporal, superior temporal and cingulated gyri from three anonymous donors were obtained from the Stanley Brain Collection (Bethesda, MD, USA). Total RNA from blood was prepared according to the manufacturer's instructions using Qiazol and RNeasy mini kit (Qiagen, Hilden, Germany). All other samples were prepared with the RNeasy mini kit (Qiagen) and prepared on a QIAcube (Qiagen). Total RNA was treated with DNase I (Invitrogen) followed by first strand cDNA synthesis using oligo-d(T)_12-18 _primers and Superscript II reagents (Invitrogen) as previously described [29].

### Real-time PCR and Tm analysis

Realtime PCR was run on an ABI Prism 7000 SDS (Applied Biosystems) with a Precision Plate Holder (Applied Biosystems, Foster City, CA, USA), white Thermo-Fast^® ^96 Detection Plates (ABgene, Epsom, UK) and the version 1.2.3 SDS software package (Applied Biosystems). Platinum SYBR Green qPCR SuperMix UDG (Invitrogen) was used in each of the 25 μl reactions containing 250 nM of forward (TCAGGTCAACAATAGGATGACAACA) and reverse (CAATGAGGGTCTACACTGGGAACT) primers (Invitrogen) directed at the HERV-W *gag *gene and 133 nM of the Tm probe (Eurogentech, Seraing, Belgium) as previously described [29]. Samples of cDNA were added in a dilution containing approximately one template molecule per reaction. Melting temperature profiles observed with more than one peak, a broader peak than expected from one sequence or no peaks were excluded. Tm data was generated with GcTm http://www.neuro.ki.se/kristensson/Tmanalysis.html as previously described [29]. Tm data was analyzed by mixture models analysis as previously described [30].

Distances between tissues were calculated, based on variance in mixing proportions, as the sum of the square of the difference, for every Tm category, of the mixing proportion between a tissue and the average of tissues.

Phylogenies were inferred from Pearson's correlation coefficient (r) of frequency distributions of Tm's with the PHYLIP (Phylogeny Inference Package) version 3.6 [40]. The neighbor joining method was used to group averages of tissue samples based on pair-wise comparisons of (1-r).

### Statistics

Data on frequency distributions into Tm categories (mixing proportions) in different tissues was compared by Chi-squared tests (GraphPad Prism 3.02, GraphPad Software, Inc., San Diego, CA, USA). Differences between distances were analyzed with the Mann-Whitney U test, p < 0.01 was considered significant.

## Authors' contributions

CN conceived the study, performed the analyses and prepared the manuscript, FL performed analyses and critically revised the manuscript, FU developed the mixture models analysis method and critically revised the manuscript, JT developed the mixture models analysis method and critically revised the manuscript, HK conceived the study and prepared the manuscript.
